# Supplementary material for: Analysis of CircRNA Expression in Peripheral Blood of Holstein Cows in Response to Heat Stress
Source: Int J Mol Sci. 2023 Jun 15;24(12):10150. doi: 10.3390/ijms241210150 (PMC10299673; doi:10.3390/ijms241210150)
Supplement: Supplementary file 1 [file ijms-24-10150-s001.zip › Table S10.pdf]

Supplementary files 10 Composition and nutrient levels of basal diets

Ingredient	Dry matter basis (%)
Corn grain	28.70
Wheat bran	5.30
Soybean meal	6.40
Cottonseed meal	8.00
Chinese wild rye	25.00
Alfalfa hay	25.00
Calcium hydrogen phosphate	0.55
Mineral and vitamin premix [1]	0.50
Salt	0.55
**Nutrient composition**	**Dry matter basis (%)**
NE_L ($MJ\cdot kg^{-1}$) [2]	6.48 $MJ\cdot kg^{-1}$
Crude protein	15.40
Neutral detergent fiber	39.60
Acid detergent fiber	21.70
Ca	0.80
P	0.46

Note: [1] Contained (per kilogram) vitamin A 9.5×10^5 IU, vitamin D 4×10^4 IU, vitamin E 7.5×10^3 IU, Fe 3×10^3 mg, Cu 2×10^3 mg/kg, Mn 2.5×10^3 mg, Zn 8×10^3 mg, Co 20 mg, I 2100 mg and Se 60 mg. [2] NE_L is calculated based on the composition of raw materials. The rest are measured values.
