# Supplementary material for: Analysis of CircRNA Expression in Peripheral Blood of Holstein Cows in Response to Heat Stress
Source: Int J Mol Sci. 2023 Jun 15;24(12):10150. doi: 10.3390/ijms241210150 (PMC10299673; doi:10.3390/ijms241210150)
Supplement: Supplementary file 1 [file ijms-24-10150-s001.zip › Table S4 34 overlap circRNAs.pdf]

Accession	Source gene	Sum1 vs Spr1			Sum1 vs Spr2		
		log_2(Fold Change)	regulation	p-value	log_2(Fold Change)	regulation	p-value
circRNA27512	ASPH	inf	up	0.00000020	inf	up	0.00000019
circRNA4902	NRDC	-inf	down	0.00000045	-inf	down	0.00065071
circRNA10995	PHF21A	-inf	down	0.00019047	-inf	down	0.00019583
circRNA14518	TXNDC16	-inf	down	0.00160854	-inf	down	0.00007698
circRNA5456	ATP2C1	-inf	down	0.00223572	-inf	down	0.01251287
circRNA15594	THADA	-inf	down	0.00562197	inf	up	0.00706730
circRNA27332	RALGPS2	inf	up	0.00832166	inf	up	0.00706730
circRNA4205	NR3C1	-inf	down	0.01468864	-inf	down	0.00001445
circRNA10586	TMBIM4	-inf	down	0.01473002	-inf	down	0.02690126
circRNA27437	PPHLN1	inf	up	0.01656765	inf	up	0.016459023
ciRNA292	RUNX2	-inf	down	0.01784709	-inf	down	0.00337421
circRNA27346	UTRN	inf	up	0.03012005	inf	up	0.02900293
circRNA27436	TOX	inf	up	0.03165165	inf	up	0.02780933
circRNA11103	STAM2	-inf	down	0.03274586	-inf	down	0.03079113
circRNA5135	RAPGEF2	-inf	down	0.03818905	-inf	down	0.03404894
circRNA7726	DNAH12	2.0048627	up	0.03838692	2.8454457	up	0.04137403
ciRNA3301	WWOX	inf	up	0.04618363	inf	up	0.04168654
circRNA27501	ZFYVE16	inf	up	0.04699043	inf	up	0.04293164
circRNA4661	intergenic_circRNA	-inf	down	0.00029191	-inf	down	0.00004687
circRNA4775	intergenic_circRNA	-inf	down	0.00032880	-inf	down	0.00018568

circRNA2191	PAPOLA	-inf	down	0.00165264	-inf	down	0.00725336
circRNA27814	STXBP4	inf	up	0.00334190	inf	up	0.00302413
circRNA14780	USP34	4.9968493	up	0.00361965	2.7119713	up	0.02160151
circRNA4794	intergenic_circRNA	-inf	down	0.00693102	-inf	down	0.01037451
circRNA13493	RHEB	-inf	down	0.00960183	-inf	down	0.03735667
circRNA27860	ANKRD27	inf	up	0.01203971	inf	up	0.01106126
circRNA21830	DIAPH3	inf	up	0.01347447	3.5030191	up	0.04149689
circRNA3628	MCF2L2	-inf	down	0.01715318	-inf	down	0.03350948
circRNA14320	intergenic_circRNA	3.8734251	up	0.02974030	6.0252488	up	0.01301667
circRNA29605	ADAM19	inf	up	0.03385933	inf	up	0.03341000
circRNA27183	DOCK7	inf	up	0.03413498	inf	up	0.03061035
circRNA4603	intergenic_circRNA	-inf	down	0.03557018	-inf	down	0.00593166
circRNA5677	GPCPD1	-inf	down	0.04018036	-inf	down	0.02418185
circRNA26716	YTHDC2	inf	up	0.04573028	inf	up	0.04601907

Inf: circRNA was not expressed in either the Spr or the Sum.

GO Term	circRNAs (Source Gene)
Guanyl-nucleotide exchange factor activity	circRNA27860 (ANKRD27); circRNA27332 (RALGPS2); circRNA3628 (MCF2L2); circRNA27183 (DOCK7)
Small GTPase mediated signal transduction	circRNA27332 (RALGPS2); circRNA13493 (RHEB); circRNA27183 (DOCK7)
Repressing transcription factor binding	ciRNA292 (RUNX2); circRNA4205 (NR3C1);
Cytosol	circRNA27437 (PPHLN1); circRNA4205 (NR3C1); circRNA11103 (STAM2); circRNA13493 (RHEB); ciRNA3301 (WWOX)
**KEGG Term**	**circRNAs (Source Gene)**
Choline metabolism in cancer	circRNA13493 (RHEB); circRNA5677 (GPCPD1)
Endocytosis	circRNA11103 (STAM2); circRNA27501 (ZFYVE16)
Longevity regulating pathway	circRNA13493 (RHEB)
